# Protective Effect of *Camellia japonica* Extract on 2,4-Dinitrochlorobenzene (DNCB)-Induced Atopic Dermatitis in an SKH-1 Mouse Model

**DOI:** 10.3390/ijms26157286

**Published:** 2025-07-28

**Authors:** Chaodeng Mo, Md. Habibur Rahman, Thu Thao Pham, Cheol-Su Kim, Johny Bajgai, Kyu-Jae Lee

**Affiliations:** 1 Department of Convergence Medicine, Wonju College of Medicine, Yonsei University, Wonju 26426, Republic of Korea; chaodengmo@gmail.com (C.M.); globaldreamer1990@gmail.com (M.H.R.); phamthuthaoytcc@gmail.com (T.T.P.); cs-kim@yonsei.ac.kr (C.-S.K.); 2 Department of Global Medical Science, Yonsei University Graduate School, Wonju College of Medicine, Wonju 26426, Republic of Korea; 3 Department of Laboratory Medicine, Wonju College of Medicine, Yonsei University, Wonju 26426, Republic of Korea

**Keywords:** atopic dermatitis, *Camellia japonica* extract, anti-inflammatory, immunomodulatory, oxidative stress, antioxidant enzyme

## Abstract

Atopic dermatitis (AD) is a common chronic inflammatory skin disorder characterized by immune dysregulation and skin barrier impairment. This study evaluated the anti-inflammatory and immunomodulatory effects of *Camellia japonica* extract in a 2,4-dinitrochlorobenzene (DNCB)-induced AD mouse model using SKH-1 hairless mice. Topical application of *Camellia japonica* extract for four weeks significantly alleviated AD-like symptoms by reducing epidermal thickness, mast cell infiltration, and overall skin inflammation. Hematological analysis revealed a marked decrease in total white blood cell (WBC) and neutrophil counts. Furthermore, the *Camellia japonica* extract significantly decreased oxidative stress, as evidenced by reduced serum reactive oxygen species (ROS) and nitric oxide (NO) levels, while enhancing the activity of antioxidant enzymes such as catalase. Importantly, allergic response markers including serum immunoglobulin E (IgE), histamine, and thymic stromal lymphopoietin (TSLP), were also downregulated. At the molecular level, *Camellia japonica* extract suppressed the expression of key pro-inflammatory cytokines, including tumor necrosis factor-alpha (TNF-α), interleukin (IL)-1β, and T helper 2 (Th2)-type cytokines such as IL-4 and IL-5, while slightly upregulating the anti-inflammatory cytokine IL-10. Collectively, these findings suggest that *Camellia japonica* extract effectively modulates immune responses, suppresses allergic responses, attenuates oxidative stress, and promotes skin barrier recovery. Therefore, application of *Camellia japonica* extract holds the promising effect as a natural therapeutic agent for the prevention and treatment of AD-like skin conditions.

## 1. Introduction

Atopic dermatitis (AD) is the most common inflammatory skin disease, with oozing, pruritic, erythematous patches, and papules that lead to excoriation and serous exudates [1]. Its global prevalence has markedly increased, impacting up to 20% and 10% of children and adults, respectively [2]. In individuals with atopy, AD often precedes the development of other allergic diseases, including asthma, allergic rhinoconjunctivitis, and food allergies [3]. Systemic atopic progression can significantly reduce quality of life [4].

AD pathogenesis is complex and multifactorial, involving an interplay of immune dysregulation, skin barrier defects, genetic susceptibility, microbial dysbiosis, and environmental triggers [5]. Type 2 immune responses are crucial for both barrier breakdown and the induction of chemokines. For example, various cytokines, such as tumor necrosis factor-alpha (TNF-α), interleukin (IL)-4, IL-5, IL-13, IL-22, and IL-31, are released by Th2 cells when they connect with and are activated by epidermal dendritic cells (DCs) or group 2 innate lymphoid cells (ILC2s) [6,7]. These cytokines induce the activation and degranulation of mast cells, eosinophils, and acidophilic granulocytes, while promoting immunoglobulin E (IgE) production by B cells [5]. Thymic stromal lymphopoietin (TSLP) and histamine levels are also increased in AD lesions due to Th2-driven inflammation [8]. However, emerging data indicate that AD pathogenesis extends beyond Th2 pathways [9,10], with oxidative stress contributing significantly.

Allergens, skin dysbiosis, pollutants, and UV light are some of the factors that cause keratinocytes and immune cells to produce excessive amounts of reactive oxygen species (ROS) [11,12]. This disrupts the redox balance, damaging cellular lipids and DNA, and contributing to skin barrier dysfunction and chronic inflammation in AD [11]. Oxidative stress-related biomarkers, including reactive nitrate (NO_2_^–^), malondialdehyde (MDA), and ROS, are markedly increased in AD [11,13], while antioxidants, such as glutathione peroxide (GPx), catalase (CAT), superoxide dismutase (SOD), and glutathione (GSH), may help control these effects [14,15]. This has raised interest in natural chemicals derived from plants because of their possible safety and effectiveness in treating AD [16].

*Camellia japonica*, a member of the Theaceae family and native to China, Korea, and Japan, is valued for its medicinal and cosmetic benefits. With more than 32,000 registered cultivars [17], it contains diverse bioactive compounds—including phenolics, terpenoids, fatty acids, pigments, and biosugars—that offer antioxidant, antimicrobial, anti-inflammatory, anticancer, and anti-aging benefits [18,19,20,21]. Its high phenolic content is primarily responsible for its strong antioxidant effect, and aqueous leaf extracts show dose-dependent efficiency [22]. *Camellia japonica* ethanol leaf and flower extracts have demonstrated efficacy against gram-positive and gram-negative bacteria [23,24], fungi (e.g., *Candida albicans* and *Cryptococcus neoformans* [24]), and viruses [25]. Moreover, by inhibiting pro-inflammatory enzymes and signaling pathways such as p38 mitogen-activated protein kinase (MAPK) and extracellular signal-regulated kinase (ERK) [26], *Camellia japonica* has anti-inflammatory properties and promotes wound healing [27,28]. These attributes make *Camellia japonica* a promising natural ingredient in biomedical and cosmetic applications.

Considering its traditional uses and documented pharmacological activities, we hypothesized that *Camellia japonica* could alleviate 2,4-dinitrochlorobenzene (DNCB)-induced AD by reducing oxidative stress, modulating inflammatory cytokine and antioxidant enzyme activities, and regulating T-cell-mediated immune responses. However, its therapeutic effects and the mechanisms in this model remain largely unexplored. Therefore, we investigated the protective effects and underlying molecular mechanisms of *Camellia japonica* in DNCB-induced AD-like skin lesions in hairless mice.

## 2. Results

### 2.1. Effects of Camellia japonica Extract on AD-like Skin Lesions

**Figure 1:**
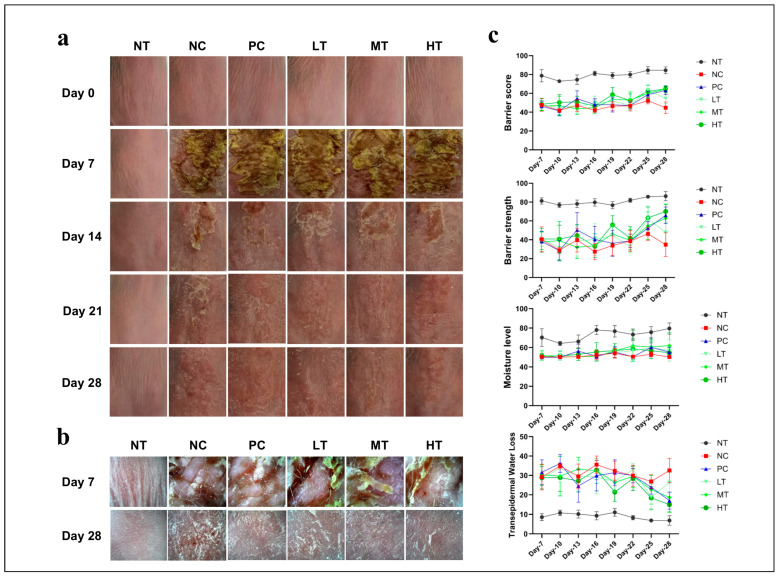
Effects of *Camellia japonica* extract on DNCB-induced AD-like clinical symptoms in SKH-1 mice. (**a**) Representative photographs of the dorsal skin in each group. (**b**) Magnified representative images of the dorsal skin on the first and last day of treatment after DNCB induction in each treatment group. (**c**) Skin condition parameters in each group. Data are presented as the mean ± standard deviation (SD). NT, no treatment group; NC, negative control group; PC, positive control group; LT, low-concentration 4% extract treatment group; MT, mid-concentration 20% extract treatment group; HT, high-concentration 100% extract treatment group.

To investigate the protective effects of *Camellia japonica* extract on AD-like skin inflammation, the mice were treated with topical DNCB application to the dorsal skin for one week to induce lesions. Over four weeks, the skin condition was monitored. One week after the DNCB application, AD-like skin lesions appeared, characterized by dryness, epidermal peeling, redness, bleeding, ulceration, and scaling. Within three weeks of localized *Camellia japonica* extract application, the experimental group showed significant improvement (Figure 1a). Compared with the NC group, the PC and *Camellia japonica* extract-treated groups exhibited reduced skin bleeding and scaling (Figure 1b) alongside improved skin barrier function and strength, moisture levels, and TEWL scores compared to the NC group (Figure 1c). No significant differences were observed between the PC group and the *Camellia japonica* extract-treated group.

### 2.2. Effects of Camellia japonica Extract on AD-like Skin Lesions Investigated Through Histopathological Analysis

**Figure 2:**
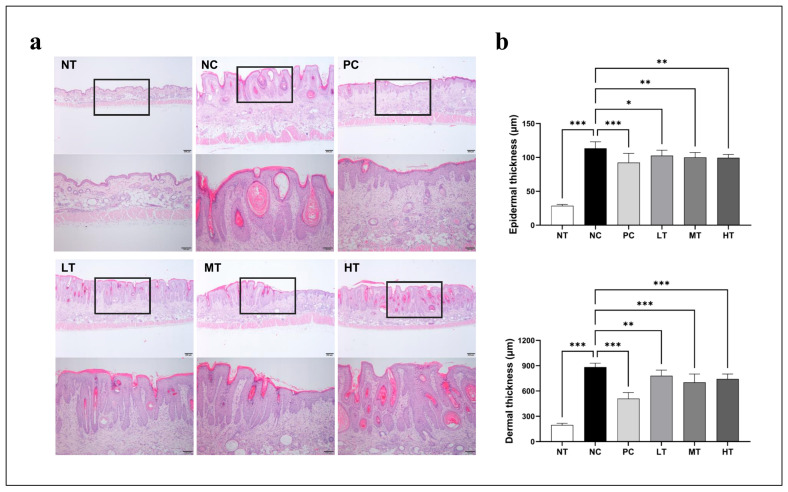
Effects of *Camellia japonica* extract on dermal and epidermal thickness. (**a**) Representative photomicrographs of dorsal skin sections stained with H&E (scale bar, 100 µm and 200 µm). (**b**) Epidermal and dermal thicknesses in H&E-stained tissue microphotographs. Data are presented as the mean ± standard deviation (SD); analysis of variance (ANOVA) and Tukey’s test. * *p* < 0.05, ** *p* < 0.01, *** *p* < 0.001 vs. NC. NT, no treatment group; NC, negative control group; PC, positive control group; LT, low-concentration 4% extract treatment group; MT, mid-concentration 20% extract treatment group; HT, high-concentration 100% extract treatment group.

To analyze the histological changes in the skin lesions following the therapy, the mice’s dorsal skin was isolated and stained with H&E. Following the DNCB treatment, the dorsal skin showed hyperkeratosis, epidermal and dermal thickening, and inflammatory cell accumulation. However, these effects were significantly inhibited by the application of the tacrolimus ointment and *Camellia japonica* extract (Figure 2a). Compared to the NC group, the epidermal and dermal thicknesses in the PC and *Camellia japonica* extract-treated groups were significantly reduced (Figure 2b).

### 2.3. Effects of Camellia japonica Extract on Systemic Immune Cell

**Figure 3:**
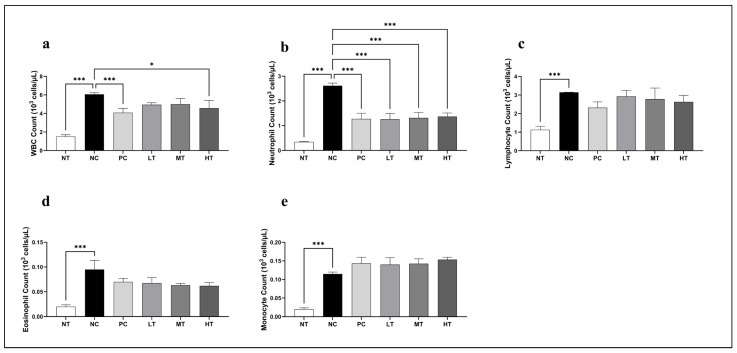
Effects of *Camellia japonica* extract on total and differential leukocyte counts. (**a**) Total WBC count. (**b**) Neutrophil count. (**c**) Lymphocyte count. (**d**) Eosinophil count. (**e**) Monocyte count. Data are presented as the mean ± Standard deviation (SD); analysis of variance (ANOVA) and Tukey’s test. * *p* < 0.05, *** *p* < 0.001 vs. NC. NT, no treatment group; NC, negative control group; PC, positive control group; LT, low-concentration 4% extract treatment group; MT, mid-concentration 20% extract treatment group; HT, high-concentration 100% extract treatment group.

The total WBC count and differential leukocyte profiles were analyzed to evaluate the immunomodulatory effects of the *Camellia japonica* extract (Figure 3). Treatment with the extract significantly decreased the total WBC and neutrophil counts, comparable to those observed in the NC group. Additionally, lymphocyte and eosinophil counts showed a moderate decline, whereas monocyte levels showed a modest elevation compared to the NC group. Hence, the *Camellia japonica* extract may exert regulatory effects on systemic immune responses, particularly by modulating neutrophil-mediated inflammation.

### 2.4. Effects of Camellia japonica Extract on Oxidative Stress Profiles

**Figure 4:**
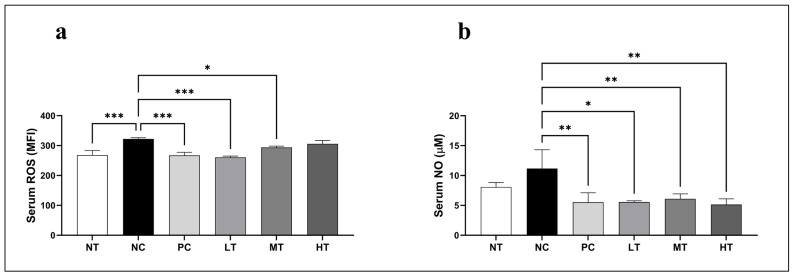
Alterations in serum ROS and NO levels after *Camellia japonica* extract treatment in a DNCB-induced AD mouse model. (**a**) Serum ROS levels. (**b**) Serum NO levels. Data are presented as the mean ± standard deviation (SD); analysis of variance (ANOVA) and Tukey’s test. * *p* < 0.05, ** *p* < 0.01, *** *p* < 0.001 vs. NC. NT, no treatment group; NC, negative control group; PC, positive control group; LT, low-concentration 4% extract treatment group; MT, mid-concentration 20% extract treatment group; HT, high-concentration 100% extract treatment group.

To evaluate the effects of the *Camellia japonica* extract on DNCB-induced oxidative stress, serum ROS and NO levels were measured in the SKH-1 hairless mice. The NC group had considerably greater serum ROS levels than the NT group (Figure 4a). Treatment with the *Camellia japonica* extract significantly lowered ROS levels when compared to the control group. Similarly, serum NO levels were significantly greater in the NC group compared to the NT group. Treatment with the *Camellia japonica* extract significantly reduced NO levels compared to the NC group (Figure 4b). These results demonstrate that the *Camellia japonica* extract reduces key oxidative stress markers.

### 2.5. Effects of Camellia japonica Extract on Antioxidative Enzymatic Activities

**Figure 5:**
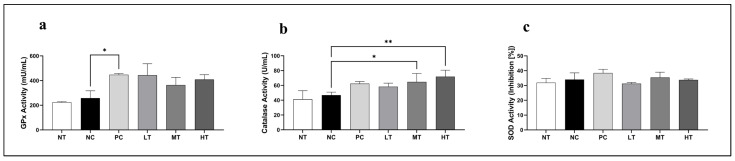
Effects of *Camellia japonica* extract on total antioxidant enzyme activities in a DNCB-induced AD mouse model. (**a**) GPx activity. (**b**) CAT activity. (**c**) SOD activity. Data are presented as the mean ± standard deviation (SD); analysis of variance (ANOVA) and Tukey’s test. * *p* < 0.05, ** *p* < 0.01 vs. NC. NT, no treatment group; NC, negative control group; PC, positive control group; LT, low-concentration 4% extract treatment group; MT, mid-concentration 20% extract treatment group; HT, high-concentration 100% extract treatment group.

The effects of the *Camellia japonica* extract on key antioxidant enzyme activity were investigated in the SKH-1 mice with DNCB-induced AD-like skin lesions. Significant alterations were found in GPx, CAT, and SOD activity. Serum GPx activity was significantly higher in the PC group than in the NC group, with similar increases observed in other *Camellia japonica* extract-treated groups (Figure 5a). In addition, CAT activity was significantly upregulated in the MT and HT treatment groups compared to the NC group (Figure 5b). No significant differences were seen in any of the treatment groups. Overall, our findings indicate that the *Camellia japonica* extract has antioxidant properties by increasing GPx and CAT activities.

### 2.6. Effects of Camellia japonica Extract on Key Allergy-Associated Biomarkers

**Figure 6:**
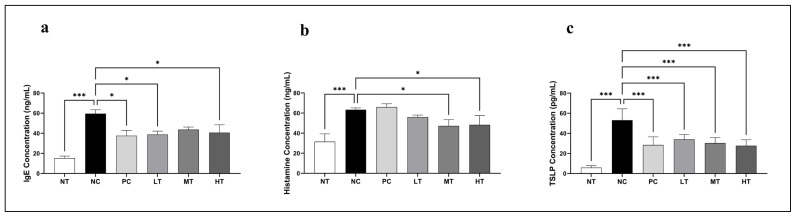
Effects of *Camellia japonica* extract on (**a**) IgE, (**b**) histamine, and (**c**) TSLP levels in mice with DNCB-induced AD. Data are presented as the mean ± standard deviation (SD); analysis of variance (ANOVA) and Tukey’s test. * *p* < 0.05, *** *p* < 0.001 vs. NC. NT, no treatment group; NC, negative control group; PC, positive control group; LT, low-concentration 4% extract treatment group; MT, mid-concentration 20% extract treatment group; HT, high-concentration 100% extract treatment group.

To assess the immunomodulatory effects of the *Camellia japonica* extract on major allergic inflammatory mediators associated with AD, serum IgE, histamine, and TSLP levels were measured in the SKH-1 mice with DNCB-induced AD-like skin lesions. The NC group exhibited significantly elevated levels of IgE, histamine, and TSLP compared with the NT group, confirming the successful induction of AD-like inflammation (Figure 6a). Treatment with the *Camellia japonica* extract notably reduced serum IgE levels, with significant decreases in the LT and HT groups compared with the NC group. Similarly, histamine levels were suppressed, particularly in the MT and HT groups (Figure 6b). Notably, TSLP expression was significantly downregulated in all *Camellia japonica* extract-treated groups, reaching levels comparable to those in the PC group (Figure 6c). Hence, *Camellia japonica* extract might alleviate AD-like symptoms by suppressing IgE, histamine, and TSLP.

### 2.7. Effects of Camellia japonica Extract on Serum Pro-Inflammatory, Th1 and Th2 Cytokine Profiles

**Figure 7:**
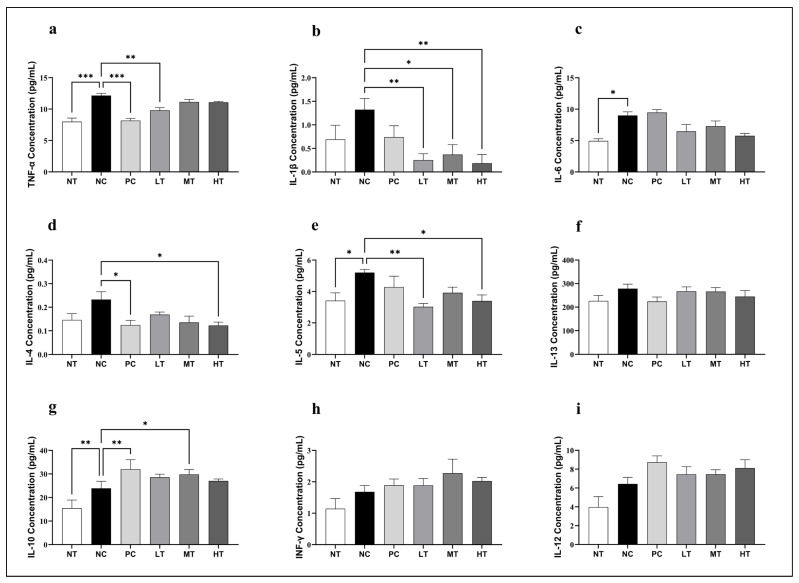
Effects of *Camellia japonica* extract on serum pro-inflammatory, Th1, and Th2 cytokine levels in mice with DNCB-induced AD-like skin lesions. Serum concentrations of (**a**) TNF-α, (**b**) IL-1β, (**c**) IL-6, (**d**) IL-4, (**e**) IL-5, (**f**) IL-13, (**g**) IL-10, (**h**) IFN-γ, and (**i**) IL-12 were measured. Data are presented as the mean ± standard deviation (SD). Significant differences were analyzed with ANOVA and Tukey’s test. * *p* < 0.05, ** *p* < 0.01, *** *p* < 0.001 vs. NC. NT, no treatment group; NC, negative control group; PC, positive control group; LT, low-concentration 4% extract treatment group; MT, mid-concentration 20% extract treatment group; HT, high-concentration 100% extract treatment group.

To investigate the immunoregulatory properties of the *Camellia japonica* extract in the AD-like skin lesions, serum cytokine levels were measured in the SKH-1 mice exposed to DNCB (Figure 7). In contrast to the NT group, the NC group’s TNF-α, IL-1β, and IL-6 levels were significantly higher, indicating increased systemic inflammation. TNF-α levels were considerably lower in the PC and LT groups after treatment with the *Camellia japonica* extract than in the NC group. IL-1β expression was strongly suppressed across all treatment groups, with the most substantial decrease in the HT group. Likewise, there were no appreciable variations in the dose-dependent decrease of IL-6 levels after extract administration. The NC group had higher levels of the Th2-associated cytokines IL-4, IL-5, and IL-13. While IL-13 showed a drop across all treatment groups with no discernible changes, the *Camellia japonica* extract successfully reduced IL-4 and IL-5 levels in all treatment groups, especially in the HT group. On the other hand, all groups treated with the *Camellia japonica* extract showed a significant upregulation of the anti-inflammatory cytokine IL-10, with the MT group exhibiting the highest expression. Moreover, the Th1 cytokines IFN-γ and IL-12 were downregulated in the NC group and moderately restored following *Camellia japonica* extract treatment, suggesting a partial rebalancing of the Th1/Th2 immune responses. These findings demonstrate that the *Camellia japonica* extract suppresses key inflammatory mediators while promoting regulatory cytokines.

## 3. Discussion

This study demonstrates the therapeutic potential of *Camellia japonica* extract to ameliorate DNCB-induced AD in SKH-1 hairless mice by improving skin barrier function and immune redox balance. Notably, application of the extract improved TEWL and epidermal thickness while suppressing allergic indicators, highlighting its role as a natural barrier enhancer. Disruption of the skin barrier plays a central role in the pathogenesis of the disease [29]. Hence, the bioactive components of the *Camellia japonica* extract promoted recovery from skin barrier dysfunction by improving moisture retention and supporting tissue regeneration. These results align with other studies that have noted the benefits of *Camellia japonica* extract in maintaining skin moisture, enhancing antioxidant activity, reducing inflammation, and promoting skin repair [30].

Previous studies have reported the anti-inflammatory and anti-allergic effects of *Camellia japonica* oil, particularly in asthma models. *Camellia japonica* oil and its major component, oleic acid, have been shown to suppress Th2-mediated inflammation by downregulating IL-4 and IL-5 as well as reducing eosinophil infiltration and IgE levels in serum [31]. These findings are consistent with our results and further support the potential of *Camellia japonica* extract as a therapeutic agent for allergic inflammatory diseases such as AD. In addition, *Camellia japonica* extract has shown anti-inflammatory and antioxidant effects in various disease models by reducing pro-inflammatory cytokines and oxidative stress [27,32]. While similar effects were observed in ocular surface diseases, including dry eye, through the reduction of ROS and inflammatory mediators [33], these studies did not specifically explore its role in AD-related immune dysregulation. Our study, therefore, addresses this gap by demonstrating that *Camellia japonica* extract not only enhances skin barrier function but also regulates systemic immune and allergic responses in an AD model.

AD is characterized by epidermal hyperplasia, increased keratin production, intracellular edema, and the accumulation of lymphocytes and mast cells [34]. Thus, we histologically evaluated dorsal epidermal thickness, cell proliferation, hyperkeratosis, spongiosis, and epidermal hyperplasia. *Camellia japonica* extract application significantly reduced the epidermal thickness in DNCB-induced AD-like skin lesions, suggesting it can effectively alleviate skin inflammation and structural changes associated with AD, protect the skin barrier, and prevent further damage.

To elucidate the immunomodulatory mechanisms underlying the expedited healing process facilitated by the *Camellia japonica* extract in DNCB-induced AD-like lesions, WBC and differential counts were performed. A notable reduction in leukocyte and differential counts was observed in the *Camellia japonica* extract-treated groups compared with the DNCB-only group, with a particularly significant decrease in the total WBC count. The substantial decline in neutrophils, lymphocytes, and eosinophils demonstrates the potent anti-inflammatory properties of the *Camellia japonica* extract in mitigating AD progression [35] and its potential to ameliorate associated immune dysfunction.

Oxidative stress and low antioxidant levels are linked to allergic skin inflammation [36,37,38,39], with ROS triggering inflammation, oxidative stress, allergic reactions, and skin barrier damage [40]. Additionally, RNS play a crucial role in the resolution of allergic inflammation and are implicated in the pathogenesis of various inflammatory disorders, including AD [41]. Remarkably, the *Camellia japonica* extract significantly lowered ROS and NO levels in mouse serum compared to the NC group. This suggests that the extract may exert a preventive effect on DNCB-induced skin tissue damage. Furthermore, the *Camellia japonica* extract-treated group exhibited increased levels of serum antioxidant enzymes, including GPx and catalase, compared with the NC group. This implies that higher antioxidant activity may protect against oxidative stress by scavenging peroxides.

Immunological analyses were conducted to assess the impact of *Camellia japonica* extract on SKH-1 hairless mice with DNCB-induced AD-like skin lesions. Elevated serum IgE levels, characteristic of AD, lead to greater clinical severity by increasing the release of inflammatory mediators [42]. In line with our findings on WBC counts and skin morphology, the NC group showed a significant elevation in serum IgE levels, while treatment with the *Camellia japonica* extract effectively suppressed this increase. Thus, the *Camellia japonica* extract may exert its effects via immunomodulation involving IgE pathways [43].

IgE-mediated inflammation primarily depends on its interaction with allergens, initiating immune cell activation and subsequent inflammatory mediator release [44]. Histamine, a key mediator released by mast cells and eosinophils, is integral to allergic response mechanisms [45,46]. Our findings demonstrate that, relative to the NC group, histamine levels were decreased in the *Camellia japonica* extract-treated groups. Since elevated histamine levels are linked to classic allergic symptoms, such as redness, swelling, and itching, these results suggest that the active ingredients in *Camellia japonica* extract possess anti-inflammatory properties, potentially reducing histamine by suppressing the release of pro-inflammatory cytokines. This, in turn, may attenuate cutaneous inflammation and enhance cellular function within tissues, supporting healing and regeneration.

TSLP is a critical cytokine in allergic inflammation, facilitating immune modulation, T cell differentiation, and epithelial immune responses [47]. The *Camellia japonica* extract markedly reduced TSLP concentrations compared with the NC group. Collectively, these findings suggest that the extract may ameliorate AD symptoms by enhancing immune responses to specific inflammatory stimuli.

The imbalance between Th1 and Th2 immune responses has been increasingly recognized as a contributing factor in the pathogenesis of AD, suggesting that immune dysregulation may play a pivotal role in disease development and progression [48]. During the acute phase of AD, the immune landscape is dominated by CD4^+^ Th2 cell activation, culminating in the overproduction of key cytokines, such as IL-4, IL-5, IL-10, and IL-13 [49,50]. Among these, IL-4 not only fuels inflammation but is also responsible for B-cell class switching, thereby increasing IgE production [51]. In contrast, within the chronic phase of AD, the Th1 cell-mediated immune response predominates, including IFN-γ, IL-2, and IL-12 secretion [52]. Furthermore, epidermal cells release IL-1β [53] and TNF-α [54], with substantial roles in the sensitization and elicitation phases of AD progression. The study found that *Camellia japonica* extract lowered Th2 cytokines (IL-4, IL-5, and IL-13) and increased Th1 cytokines (IFN-γ and IL-12), indicating a restored immunological balance. The equilibrium between these immune responses is essential for maintaining homeostasis under chronic inflammatory conditions. Notable reductions were also observed in pro-inflammatory cytokines (TNF-α, IL-1β, and IL-6), highlighting the anti-inflammatory properties of *Camellia japonica* extract and its ability to reduce the acute inflammatory responses associated with AD. The increase in IL-10 levels supports the role of *Camellia japonica* extract in reducing inflammation and aiding tissue healing. Overall, these results suggest that *Camellia japonica* extract may be a promising treatment for AD by helping to control immune responses and lower inflammation.

Several limitations of this study warrant consideration. First, although DNCB was used to induce AD-like lesions in mice, a widely accepted experimental model, it does not fully replicate the complex pathophysiology of human AD, which involves genetic predisposition, immune dysregulation, and skin barrier dysfunction. Therefore, attentiveness is needed to generalize these findings to human AD. Second, while the study demonstrates the anti-inflammatory and antioxidant effects of *Camellia japonica* extract, the precise molecular mechanisms underlying its therapeutic effects remain unclear and warrant further investigation. Third, biochemical markers related to oxidative stress, inflammation, and allergic responses were evaluated only in the serum, not directly in the lesional skin tissue. Therefore, future studies are needed and should focus on analyzing local skin responses and conducting detailed mechanistic investigations at the site of inflammation. Lastly, clinical translation of these results will require extensive human studies to evaluate the extract’s safety, effective dosage, and potential long-term outcomes in managing AD.

## 4. Materials and Methods

### 4.1. Preparation of Camellia japonica Extract

*Camellia japonica extracts* were supplied by Wando County, jeollanam-do, Republic of Korea. Fresh *Camellia japonica* flowers were harvested, thoroughly washed with distilled water to remove impurities, and air-dried to eliminate surface moisture. The cleaned flowers were then finely chopped, freeze-dried, and ground into a fine powder. Sugar was added to the powdered material to induce fermentation, which was carried out under controlled conditions for a specified period. After fermentation, the samples were soaked in ethanol and aged at low temperatures (2–8 °C) for approximately two weeks to facilitate the extraction of bioactive compounds. The resulting mixture was concentrated under reduced pressure, leading to the separation of oil and aqueous layers. The upper oil layer was carefully collected and filtered to obtain purified *Camellia japonica* flower oil for further use.

### 4.2. Experimental Animals

Female SKH-1 hairless mice (five weeks old, 25 ± 4.2 g) from Orient Bio, Inc. (Seongnam, Republic of Korea) were acclimated for one week in plastic cages (390 × 275 × 175 mm) with wood shavings. The animal facility maintained 22 ± 2 °C, 50 ± 10% humidity, and a 12 h light/dark cycle. The mice were provided ad libitum access to standard chow and filtered water. All experimental procedures were performed in accordance with the protocol of the Institutional Animal Care and Use Committee, Yonsei University Wonju College of Medicine (YWC-230421-1).

### 4.3. Dermatitis Induction

Forty-eight mice were randomly allocated into six groups (*n* = 8/group): (1) NT: no treatment; (2) NC: negative control (100 μL 0.5% DNCB in a 3:1 mixture of acetone and olive oil); (3) PC: positive control (100 μL 0.5% DNCB + 0.1% tacrolimus ointment; Protopic LEO Laboratories Ltd., Dublin, Ireland); (4) LT: low-concentration treatment (100 μL 0.5% DNCB + 4% *Camellia japonica* extract); (5) MT: mid-concentration treatment (100 μL 0.5% DNCB + 20% *Camellia japonica* extract); and (6) HT: high-concentration treatment (100 μL 0.5% DNCB + 100% *Camellia japonica* extract). The mice were identified using skin markers on their tails. All experiments were conducted between 0900 to 1800 to minimize environmental variance.

**Figure 8:**
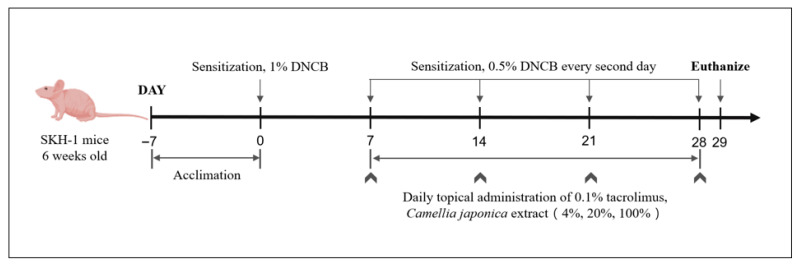
Schematic diagram of animal experimental procedure.

To establish AD-like inflammation and skin lesions, the SKH-1 hairless mice were treated with DNCB, according to previously reported studies protocols, with slight modifications [13,55]. AD was induced by applying 100 μL of 1% DNCB daily to the dorsal skin (~4 cm^2^) of all groups except the NT group for one week, followed by 100 μL of 0.5% DNCB every other day for three weeks. The experimental group received daily applications of *Camellia japonica* extract on the AD-like lesional dorsal skin, while the PC group was treated with tacrolimus ointment. The NC group received a 0.5% DNCB booster every other day, generating a stress level comparable to the PC and experimental groups without treatment. The treatments lasted three weeks (Figure 8). On days when both DNCB and treatments were administered, treatment reagents were applied 4 h after DNCB induction.

### 4.4. Evaluation of Skin Lesion Severity

Over four weeks, photographs of the dorsal region of the mice were captured every three days using a camera. Simultaneously, high-resolution images of the murine skin were acquired using an A-ONE TAB instrument (Bomtech Electronics Co., Ltd., Seoul, Republic of Korea). Assessments of skin parameters—including skin barrier score, skin barrier strength, skin moisture level, and transepidermal water loss (TEWL)—were conducted using a GP Skin device (MSIPCRM-G10 Barrier Sensor; Seoul, Republic of Korea) at 3-day intervals for a total of eight measurements. The GP Skin probe was placed on a defined area of the dorsal skin of each mouse for 10 s. The resulting data were automatically transmitted and recorded via a mobile application through Bluetooth connectivity. Each measurement was repeated thrice, and the average was calculated to ensure accuracy. This method enabled evaluation of visual and physiological skin characteristics, enabling the assessment of skin lesion severity.

### 4.5. Serum Sample Preparation

After four weeks of treatment, the mice were anesthetized with isoflurane (Hana Pharm. Co., Hwaseong, Republic of Korea) in specialized containers. Blood was collected from the retro-orbital venous plexus into BD vacutainer serum separation gel tubes with ethylenediaminetetraacetic acid (EDTA) for serum and EDTA-coated BD vacutainer tubes for white blood cell (WBC) count analysis. Immediately after blood collection, the mice were euthanized via cervical dislocation. WBC counts were assessed promptly, and serum was isolated by centrifugation at 14,000 rpm for 5 min at 4 °C. Serum protein concentration was quantified using the Pierce™ BCA Protein Assay Kit (Thermo Scientific, Rockford, IL, USA). The collected serum samples were then stored at –80 °C for subsequent analyses.

### 4.6. Histopathologic Examination

Skin samples were obtained by excising the dorsal region from each group. The tissues were fixed in 10% neutral buffered formalin (0.1 M phosphate buffer, pH 7.4) for 24 h. Following fixation, the samples were dehydrated through a graded ethanol series, cleared with xylene, and embedded in paraffin (Polysciences, Warrington, PA, USA). Thin paraffin sections, each 4 μm thick, were then prepared using a microtome (Reichert Inc., New York, NY, USA). Hematoxylin and eosin (H&E) staining was performed for histological analysis. After staining, ten fields of view were selected on each slide, and the thicknesses of the epidermis and dermis were measured at fixed-width intervals. Thickness measurements were obtained using microscopy software (Cell Sens Standard v3.2, Olympus, Tokyo, Japan), and histological changes were documented at 40× and 100× magnifications under an optical microscope (BA300; Motic Ltd., Hong Kong, China).

### 4.7. WBC and Differential Count Analysis

The retro-orbital venous plexus was used to collect blood, which was then promptly combined with an automatic roller mixer for five minutes in BD vacutainer tubes coated with EDTA. Total WBCs and differentiated lymphocytes, neutrophils, monocytes, eosinophils, and basophils were measured using a Hemavet HV950 FS automated hematology analyzer (Drew Scientific Inc., Dallas, TX, USA).

### 4.8. Detection of ROS Level in Serum

The effect of *Camellia japonica* extract on serum oxidative stress and superoxide levels was evaluated by measuring ROS using 2-4-dichlorodihydrofluorescein diacetate (DCFH-DA; Sigma, St. Louis, MO, USA), according to the manufacturer’s instructions. A 96-well black plate was filled with 5 μL of serum and 100 μL of 20 µM DCFH-DA. The mixture was then mixed and incubated for 30 min at 37 °C. With a DTX multimode plate reader (Beckman Coulter, Inc., Fullerton, CA, USA), fluorescence was measured at 488 nm excitation and 525 nm emission.

### 4.9. Detection of Serum NO Levels

The Griess reagent (Promega Corp., Madison, WI, USA) was used to test the NO levels, and the manufacturer’s instructions were followed to calculate the serum nitrite (NO_2_) concentrations. In summary, 10 μL of serum samples and 50 μL of Griess reagent (sulfanilamide and N-1-naphthyl ethylenediamine dihydrochloride) were mixed in a 96-well microplate, allowed to sit at room temperature in the dark for 10 min, and then the absorbance was measured at 550 nm using a SpectraMax^®^ ABS Plus (Molecular Devices, San Jose, CA, USA). The absorbance of the sample was compared to a nitrite standard reference curve to calculate the NO concentration.

### 4.10. Detection of Serum Antioxidant Activities

Serum antioxidant enzyme levels were assessed using Cayman assay kits (Cayman Chemical Co., Ann Arbor, MI, USA) for GPx activity and Biomax assay kits (Bio-Maxmall Co., Gyeonggi-do, Suwon, Republic of Korea) for SOD and CAT, following the manufacturer’s instructions. In summary, a 96-well microplate was filled with 5 μL of serum and the appropriate reagent. Absorbance was measured using SpectraMax^®^ ABS Plus (Molecular Devices, San Jose, CA, USA) at 450 nm (SOD), 570 nm (CAT), and 340 nm (GPx) following a 30 min incubation period. Concentrations of antioxidant enzymes were computed by comparing them to reference curves.

### 4.11. Detection of Serum Lactate Dehydrogenase Enzyme Activities

Serum lactate dehydrogenase (LDH) activity was evaluated using an Abcam assay kit (Abcam ab102526, Cambridge, UK) to evaluate cell and tissue damage. Briefly, a SpectraMax^®^ ABS Plus reader (Molecular Devices, San Jose, CA, USA) was used to measure absorbance at 450 nm following the development of colorimetric indicators in nicotinamide adenine dinucleotide (NADH) standards and experimental samples for 30 min at room temperature. As directed by the manufacturer, LDH activity was computed by comparing it to a standard reference curve.

### 4.12. Detection of Total IgE Level in Serum

The total IgE serum concentration was measured using a mouse IgE enzyme-linked immunosorbent assay (ELISA) kit (Thermo Fisher Scientific Korea Ltd., Seoul, Republic of Korea). Serum samples (10 µL) were diluted and processed according to the instructions of the manufacturer. Absorbance at 450 nm was measured using the SpectraMax^®^ ABS Plus (Molecular Devices) within 30 min following addition of the stop solution. Total IgE concentrations were calculated via linear regression analysis based on the standard absorbance values.

### 4.13. Detection of Total Serum Histamine Level

Serum total histamine concentrations were measured using a Novus Biologicals ELISA kit (Novus Biologicals, Centennial, CO, USA). Briefly, detection samples of 10 μL serum and 40 μL sample diluent were prepared according to the instructions of the manufacturer. After color development at 37 °C in the presence of the reaction substrate under light-shielded conditions, the stop solution was added, and absorbance at 450 nm was measured using a SpectraMax^®^ ABS Plus reader (Molecular Devices, San Jose, CA, USA). Total serum histamine concentrations were calculated from the standard curve and adjusted by the dilution factor.

### 4.14. Detection of Total Serum TSLP Level

Serum total TSLP concentrations were measured using a TSLP ELISA kit (R&D Systems, Minneapolis, MN, USA). For each assay, 40 μL of sample diluent was mixed with 10 μL of serum and added to the wells of a microplate. The procedure was performed according to the manufacturer’s instructions provided in the kit manual. Optical density was measured at 450 nm using a SpectraMax^®^ ABS Plus reader (Molecular Devices) within 30 min after adding the stop solution. The final TSLP concentration in the serum was calculated by multiplying the value obtained from the standard curve by the appropriate dilution factor.

### 4.15. Detection of Serum Cytokine Levels

Serum cytokines, including IL-1β, IL-4, IL-5, IL-6, IL-10, IL-12, IL-13, interferon-gamma (IFN-γ), and TNF-α, were measured using a multiplex array assay kit (2023 Merck KGaA, Darmstadt, Germany). Following the instructions of the manufacturer, the mean fluorescence intensity (MFI) was determined from microplates using Luminex technology (Bio-Plex Multiplex Bead Array System™, Bio-Rad, Hercules, CA, USA). Standard curves for each cytokine were generated according to the reference concentrations supplied with the assay kit, and cytokine concentrations were calculated utilizing a five-parameter logistic method with dedicated analysis software.

### 4.16. Statistical Analysis Procedure

All data from each experiment were analyzed and compared using two-way analysis of variance (ANOVA), with Tukey’s post hoc test for multiple comparisons. The analysis was carried out using the GraphPad Prism 10.1.2 software package (GraphPad, La Jolla, CA, USA). The statistical significance level was chosen at *p* < 0.05. Data are expressed as mean ± standard deviation (SD).

## 5. Conclusions

Collectively, our results suggest that *Camellia japonica* extract treatment may exert regional and systemic therapeutic effects against AD skin lesions by enhancing skin barrier function and modulating immune redox responses. These immunosuppressive effects may be attributed to the synergistic action of *Camellia japonica* extract-specific bioactive compounds, which possess anti-allergic and anti-inflammatory properties. Although our findings highlight the potential utility of the *Camellia japonica* extract as a pharmacological agent for AD, further studies are required to evaluate its clinical efficacy and preventive effects in patients with AD.

## Data Availability

All data analyzed in this study are included in this article.

## Abbreviations

AD	Atopic dermatitis
CAT	Catalase
DCFH-DA	2′,7′-Dichlorodihydrofluorescein diacetate
DNCB	2,4-Dinitrochlorobenzene
EDTA	Ethylenediaminetetraacetic acid
ELISA	Enzyme-linked immunosorbent assay
ERK	Extracellular signal-regulated kinase
GPx	Glutathione peroxidase
GSH	Glutathione
H&E	Hematoxylin and eosin
IFN-γ	Interferon-gamma
IgE	Immunoglobulin E
IL	Interleukin
LDH	Lactate dehydrogenase
NADH	Nicotinamide adenine dinucleotide
NIH	National Institutes of Health
NO	Nitric oxide
NO_2_^−^	Nitrite
p38 MAPK	p38 mitogen-activated protein kinase
RNS	Reactive nitrogen species
ROS	Reactive oxygen species
SD	Standard deviation
SOD	Superoxide dismutase
TEWL	Transepidermal water loss
TNF-α	Tumor necrosis factor-alpha
TSLP	Thymic stromal lymphopoietin
WBCs	White blood cells
